# Supplementary material for: Denovo Production of Resveratrol by Engineered Rice Wine Strain Saccharomyces cerevisiae HJ08 and Its Application in Rice Wine Brewing
Source: J Fungi (Basel). 2024 Jul 23;10(8):513. doi: 10.3390/jof10080513 (PMC11355211; doi:10.3390/jof10080513)
Supplement: Supplementary file 1 [file jof-10-00513-s001.zip › jof-3086807-supplementary.pdf]

Supplementary Figures

Figure S1: Map of p426-*TAL1*

Figure S2: Map of p426-*4CL*

Figure S3: Map of p426-*STS*

Figure S4: Map of p426-*TAL2*

Figure S5: HPLC analyses the titer of resveratrol in rice wine by engineered strain HJ08 supplemented with and without tyrosine

Table S1: Codon-optimized sequence of the *RtTAL*, *Pc4CL* and *VvSTS* genes

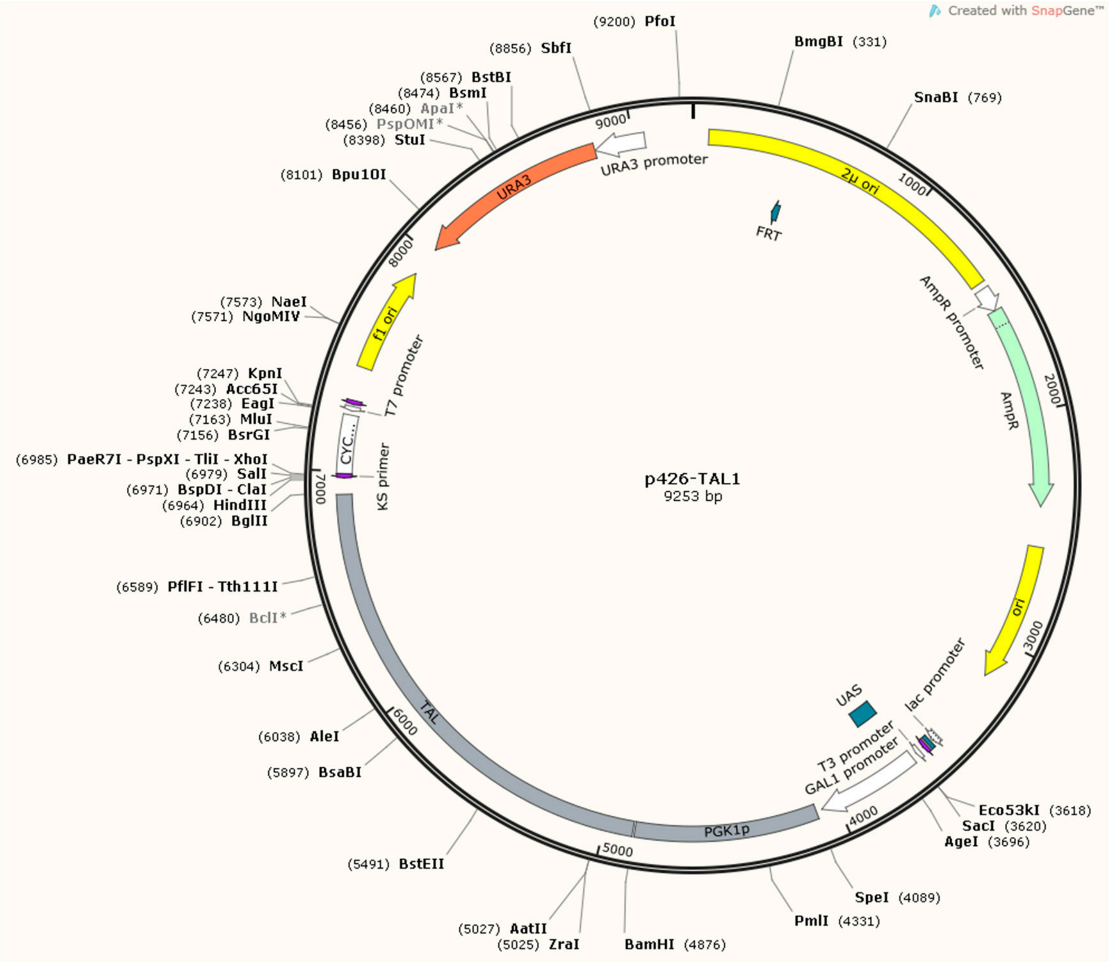
Created with SnapGene™
p426-TAL1
9253 bp
(9200) PfoI
BmgBI (331)
SnaBI (769)
(8856) SbfI
(8567) BstBI
(8474) BsmI
(8460) ApaI*
(8456) PspOMI*
(8398) StuI
(8101) Bpu10I
(7573) NaeI
(7571) NgoMIV
(7247) KpnI
(7243) Acc65I
(7238) EagI
(7163) MluI
(7156) BsrGI
(6985) PaeR7I - PspXI - TliI - XhoI
(6979) SalI
(6971) BspDI - ClaI
(6964) HindIII
(6902) BglII
(6589) PflFI - Tth111I
(6480) BclI*
(6304) MscI
(6038) AleI
(5897) BsaBI
(5491) BstEII
(5027) AatII
(5025) ZraI
BamHI (4876)
PmlI (4331)
SpeI (4089)
Eco53kI (3618)
SacI (3620)
AgeI (3696)
URA3 promoter
URA3
2μ ori
FRT
AmpR promoter
AmpR
ori
lac promoter
UAS
T3 promoter
GAL1 promoter
PGK1p
TAL
KS primer
CYC...
T7 promoter
f1 ori
1000
2000
3000
4000
5000
6000
7000
8000
9000

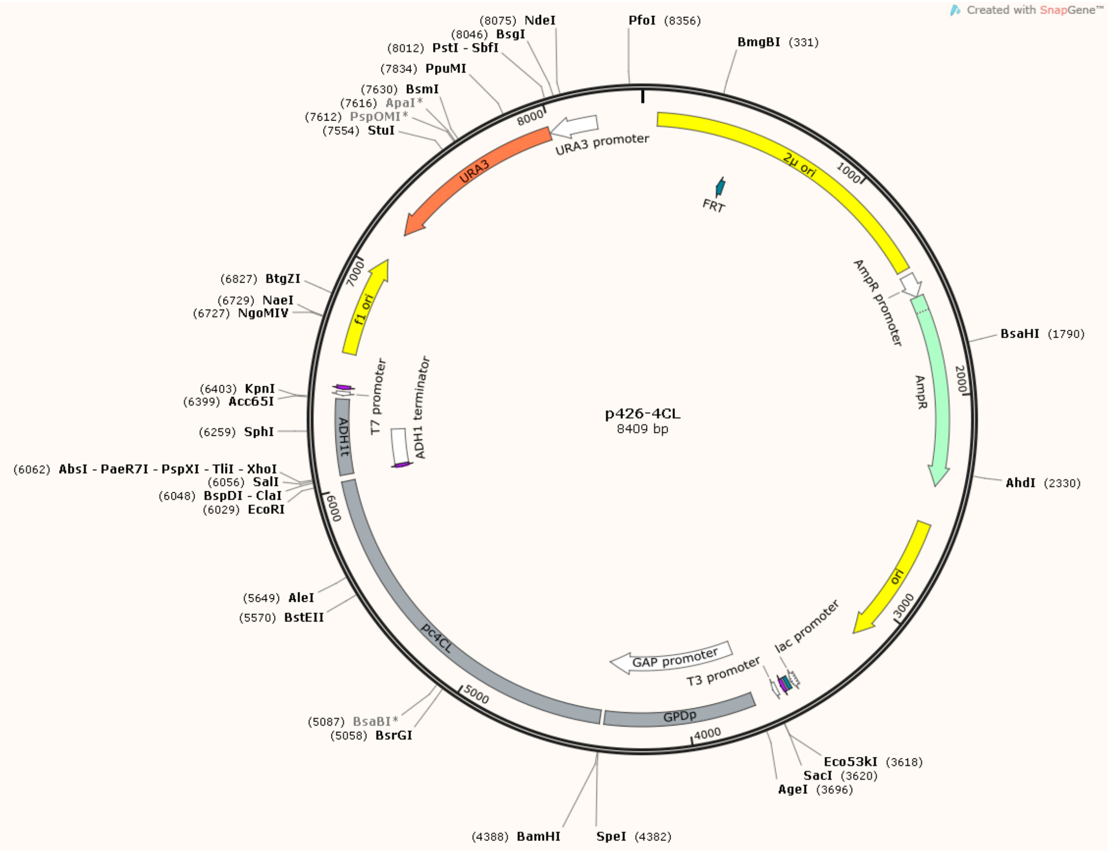
Created with SnapGene™
p426-4CL
8409 bp
(8075) NdeI
(8046) BsgI
(8012) PstI - SbfI
(7834) PpuMI
(7630) BsmI
(7616) ApaI*
(7612) PspOMI*
(7554) StuI
PfoI (8356)
BmgBI (331)
(6827) BtgZI
(6729) NaeI
(6727) NgoMIV
BsaHI (1790)
(6403) KpnI
(6399) Acc65I
(6259) SphI
(6062) AbsI - PaeR7I - PspXI - TliI - XhoI
(6056) SalI
(6048) BspDI - ClaI
(6029) EcoRI
AhdI (2330)
(5649) AleI
(5570) BstEII
(5087) BsaBI*
(5058) BsrGI
Eco53kI (3618)
SacI (3620)
AgeI (3696)
(4388) BamHI
SpeI (4382)
URA3 promoter
URA3
2μ ori
FRT
AmpR promoter
AmpR
ori
f1 ori
T7 promoter
ADH1 terminator
ADH1t
pc4CL
GPDp
GAP promoter
T3 promoter
lac promoter
1000
2000
3000
4000
5000
6000
7000
8000

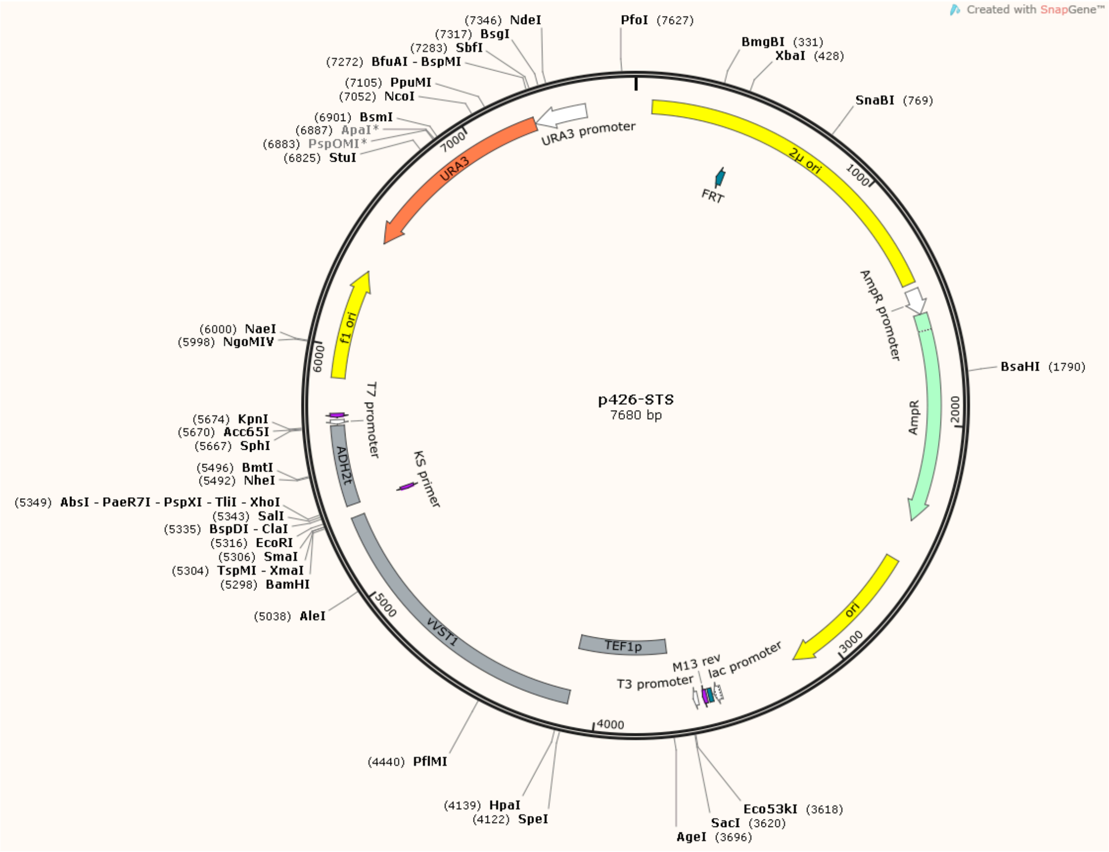

Created with SnapGene™
p426-STS
7680 bp
(7346) NdeI
(7317) BsgI
(7283) SbfI
(7272) BfuAI - BspMI
(7105) PpuMI
(7052) NcoI
(6901) BsmI
(6887) ApaI*
(6883) PspOMI*
(6825) StuI
PfoI (7627)
BmgBI (331)
XbaI (428)
SnaBI (769)
BsaHI (1790)
(6000) NaeI
(5998) NgoMIV
(5674) KpnI
(5670) Acc65I
(5667) SphI
(5496) BmtI
(5492) NheI
(5349) AbsI - PaeR7I - PspXI - TliI - XhoI
(5343) SalI
(5335) BspDI - ClaI
(5316) EcoRI
(5306) SmaI
(5304) TspMI - XmaI
(5298) BamHI
(5038) AleI
(4440) PflMI
(4139) HpaI
(4122) SpeI
Eco53kI (3618)
SacI (3620)
AgeI (3696)
URA3 promoter
URA3
2μ ori
FRT
AmpR promoter
AmpR
ori
lac promoter
M13 rev
T3 promoter
TEF1p
vVST1
ADH2t
T7 promoter
KS primer
f1 ori
1000
2000
3000
4000
5000
6000
7000

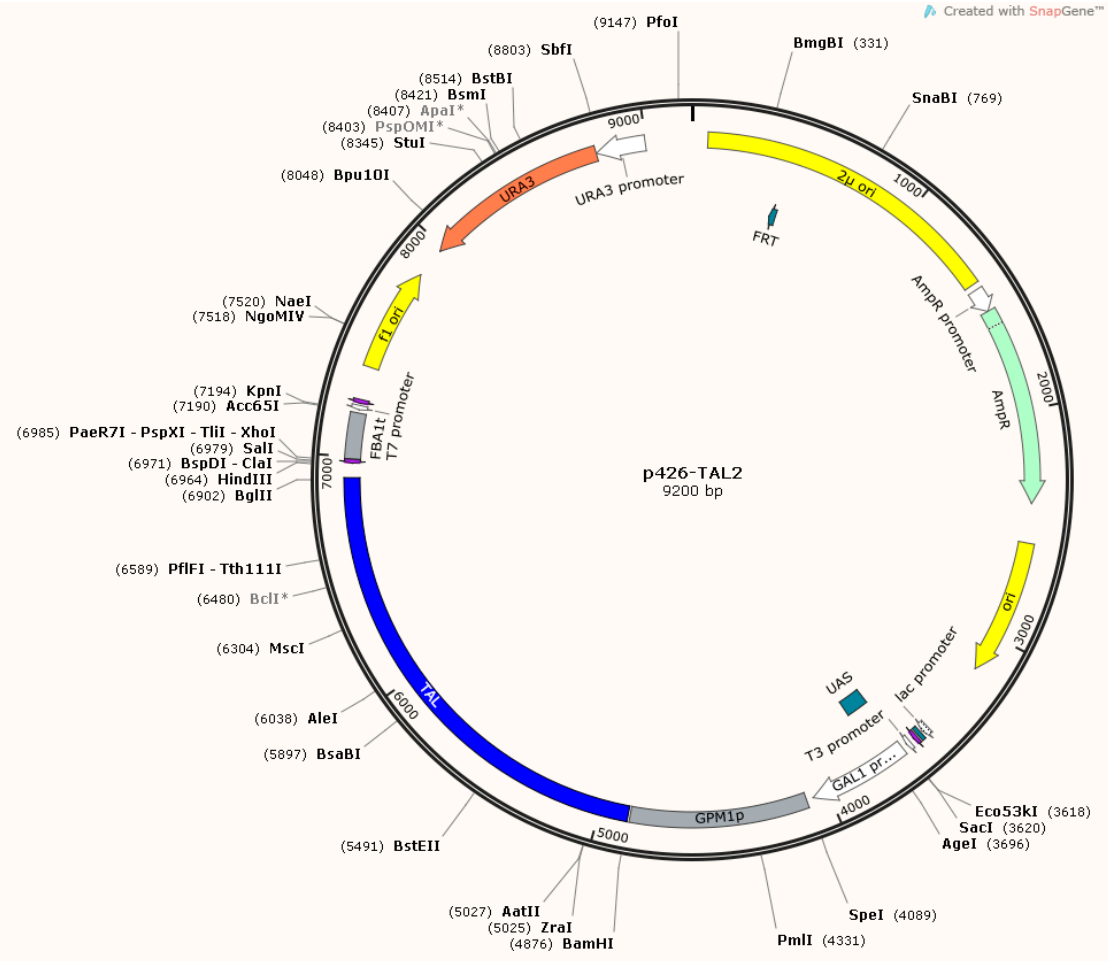

Created with SnapGene™
p426-TAL2
9200 bp
(9147) PfoI
BmgBI (331)
SnaBI (769)
(8803) SbfI
(8514) BstBI
(8421) BsmI
(8407) ApaI*
(8403) PspOMI*
(8345) StuI
(8048) Bpu10I
(7520) NaeI
(7518) NgoMIV
(7194) KpnI
(7190) Acc65I
(6985) PaeR7I - PspXI - TliI - XhoI
(6979) SalI
(6971) BspDI - ClaI
(6964) HindIII
(6902) BglII
(6589) PflFI - Tth111I
(6480) BclI*
(6304) MscI
(6038) AleI
(5897) BsaBI
(5491) BstEII
(5027) AatII
(5025) ZraI
(4876) BamHI
PmlI (4331)
SpeI (4089)
Eco53kI (3618)
SacI (3620)
AgeI (3696)
URA3
URA3 promoter
2μ ori
FRT
AmpR promoter
AmpR
ori
lac promoter
UAS
T3 promoter
GAL1 pr...
GPM1p
TAL
FBA1t
T7 promoter
f1 ori
1000
2000
3000
4000
5000
6000
7000
8000
9000

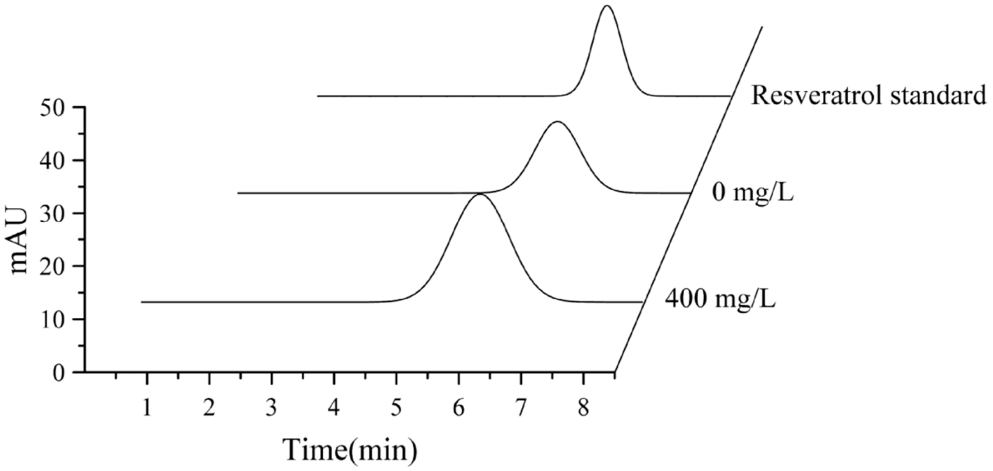

mAU
50
40
30
20
10
0
1
2
3
4
5
6
7
8
Time(min)
Resveratrol standard
0 mg/L
400 mg/L

Table S1 Genetic sequence in this study

Key gene	Sequence
RtTAL	ATGGCTCCAAGACCAACTTCTCAATCCCAAGCTAGAACTTGTC<br>CAACAACTCAAGTCACTCAAGTTGATATTGTTGAAAAGATGTT<br>AGCTGCTCCAACTGATTCTACTTTAGAATTGGATGGTTATTCTT<br>TAAACTTGGGTGACGTCGTTTCTGCTGCTAGAAAGGGTAGACC<br>AGTTAGAGTTAAGGATAGTGATGAAATTAGAAGTAAAATTGAT<br>AAATCTGTTGAATTTTTGAGATCCCAATTAAGTATGTCTGTTTAT<br>GGTGTAACTACTGGTTTTGGTGGTTCTGCTGACACTAGAACTG<br>AAGATGCTATTTCTTTGCAAAAGGCTTTGTTAGAACACCAATTA<br>TGTGGTGTTTTGCCATCTTCCTTTGATTCCTTCAGATTAGGTAG<br>AGGTTTAGAAAATTCATTGCCATTAGAAGTCGTTAGAGGTGCA<br>ATGACTATTAGAGTTAACTCTTTAACTAGAGGTCACTCTGCTGT<br>TAGATTAGTTGTCTTAGAAGCCTTGACTAACTTCCTTAATCATG<br>GTATTACTCCAATTGTCCCATTGAGAGGTACTATTTCTGCTTCTG<br>GTGACTTGTCTCCACTATCTTACATTGCTGCTGCCATTTCTGGT<br>CACCCTGATTCTAAAGTTCATGTTGTTCATGAAGGTAAAGAAA<br>AGATTTTGTACGCTAGAGAAGCTATGGCTTTGTTTAATTTGGAA<br>CCAGTTGTCTTGGGTCCAAAAGAAGGTTTAGGTTTAGTAAATG<br>GTACTGCTGTTTCCGCTTCAATGGCTACTTTAGCTTTACATGAT<br>GCACATATGTTGTCATTATTGTCTCAATCCTTGACTGCCATGACT<br>GTCGAAGCAATGGTTGGTCACGCAGGTTCTTTCCACCCATTTT<br>TGCATGATGTTACTAGACCACATCCAACTCAAATTGAAGTTGC<br>CGGTAATATTAGAAAACTATTGGAAGGTTCCAGATTTGCAGTTC<br>ACCATGAAGAAGAAGTCAAGGTTAAAGATGATGAAGGTATCTT<br>GAGACAAGATAGATATCCTTTGAGAACTTCACCACAATGGTTG<br>GGTCCATTGGTTTCCGACTTGATTCATGCTCACGCTGTGTTAAC<br>CATTGAAGCCGGTCAATCTACTACTGATAATCCATTGATTGATG<br>TTGAAAATAAGACTTCCCACCATGGTGGTAATTTTCAAGCTGC<br>AGCAGTTGCTAACACTATGGAAAAGACAAGATTGGGTTTAGCT<br>CAAATTGGTAAATTGAACTTCACTCAATTGACTGAAATGTTAA<br>ATGCCGGTATGAATAGAGGTCTACCATCATGTTTGGCCGCTGAA<br>GATCCATCATTAAGTTACCACTGTAAAGGTTTAGACATCGCCGC<br>TGCTGCCTACACCTCTGAATTAGGTCATTTGGCTAATCCTGTTA<br>CTACTCATGTTCAACCAGCTGAAATGGCCAACCAAGCTGTTAA<br>TTCACTAGCCTTGATTTCTGCTAGAAGAACTACCGAGTCTAAC<br>GATGTCTTGTCTTTGTTATTGGCAACTCATTTGTACTGTGTTTTG<br>CAAGCTATTGACTTGAGAGCTATTGAATTTGAGTTCAAGAAGC<br>AATTTGGTCCTGCCATCGTTTCCTTGATTGATCAACACTTCGGT<br>TCAGCTATGACTGGTTCTAACTTGAGAGATGAATTGGTTGAAA<br>AGGTTAACAAGACTTTAGCAAAAAGATTGGAACAAACTAATTC<br>CTACGACTTGGTCCCAAGATGGCACGATGCTTTCTCTTTTGCCG<br>CTGGTACTGTCGTTGAAGTTTTGTCTTCAACCTCTTTATCTCTA

	GCTGCTGTTAACGCATGGAAAGTTGCCGCTGCTGAATCTGCTA
	TTTCCTTAACTAGACAAGTTAGAGAAACTTTTTGGTCTGCTGC
	CTCCACCTCTTCCCCAGCTTTGTCTTATTTGTCTCCAAGAACTC
	AAATTTTGTACGCTTTCGTTAGAGAAGAGTTGGGTGTTAAGGC
	TAGAAGAGGTGACGTTTTCTTAGGTAAACAAGAAGTTACAATT
	GGTTCCAACGTCTCCAAGATCTATGAAGCTATTAAGTCTGGTAG
	AATTAACAACGTTTTGTTGAAGATGTTGGCTTAA
	ATGGGTGACTGTGTTGCTCCAAAGGAAGACTTGATCTTCAGAT
	CTAAGTTGCCAGACATCTACATCCCAAAGCACTTGCCATTGCA
	CACTTACTGTTTCGAAAACATCTCTAAGGTTGGTGACAAGTCT
	TGTTTGATCAACGGTGCTACTGGCGAAACATTCACTTACTCTC
	AGGTTGAATTGTTGTCTAGAAAGGTTGCTTCTGGTTTGAACAA
	GTTGGGTATCCAACAAGGTGACACTATCATGTTGTTGTTGCCA
	AACTCTCCAGAATACTTCTTCGCTTTCTTGGGTGCTTCTTACAG
	AGGTGCTATCTCTACTATGGCTAACCCATTCTTCACTTCTGCTG
	AAGTTATCAAGCAATTGAAGGCGAGCTTGGCTAAGTTGATAAT
	CACTCAAGCTTGTTACGTTGACAAGGTTAAGGACTACGCTGCT
	GAAAAGAACATCCAAATCATCTGTATCGACGACGCTCCACAAG
	ACTGTTTGCACTTCTCTAAGTTGATGGAAGCTGACGAATCTGA
	AATGCCAGAAGTTGTTATCGACTCTGACGATGTTGTTGCTCTCC
	CATACTCTTCGGGTACTACTGGTTTGCCAAAGGGTGTTATGTTG
	ACTCACAAGGGTTTGGTTACTTCTGTTGCTCAACAAGTTGACG
	GTGACAACCCAAACTTGTACATGCACTCTGAAGACGTTATGAT
	CTGTATCTTGCCATTGTTCCACATCTACTCTTTGAACGCTGTTTT
	GTGTTGTGGTTTGAGAGCTGGTGTTACTATCTTGATCATGCAAA
Pc4CL	AGTTCGACATCGTTCCATTCTTGGAATTGATCCAAAAGTACAA
	GGTTACTATCGGTCCATTCGTTCCACCAATCGTTTTGGCTATCG
	CTAAGTCTCCAGTTGTTGACAAGTACGACTTGTCTTCTGTTAG
	AACTGTTATGTCTGGTGCTGCTCCATTGGGTAAGGAATTGGAA
	GACGCTGTTAGAGCTAAGTTCCCAAACGCTAAGTTGGGTCAAG
	GTTACGGTATGACTGAAGCTGGTCCAGTTTTGGCTATGTGTTTG
	GCTTTCGCTAAGGAACCATACGAAATCAAGTCTGGTGCTTGTG
	GTACTGTTGTTAGAAACGCTGAAATGAAGATCGTTGACCCAGA
	AACTAACGCTTCTTTGCCAAGAAACCAAAGAGGTGAAATCTG
	TATCAGAGGTGACCAAATCATGAAGGGTTACTTGAACGACCCA
	GAATCTACTAGAACTACTATCGACGAAGAAGGTTGGTTGCACA
	CTGGTGACATCGGTTTCATCGACGACGACGACGAATTGTTCAT
	CGTTGACAGATTGAAGGAAATCATCAAGTACAAGGGTTTCCAA
	GTTGCTCCAGCTGAATTGGAAGCTTTGTTGTTGACTCACCCAA
	CTATCTCTGACGCTGCTGTTGTTCCAATGATCGACGAAAAGGC
	GGGTGAAGTTCCAGTTGCGTTCGTTGTTCGTACTAACGGCTTC
	ACTACTACTGAGGAAGAAATCAAGCAATTCGTTTCTAAGCAAG
	TTGTTTTCTACAAGAGAATCTTCAGAGTTTTCTTCGTTGACGCT
	ATCCCAAAGTCTCCATCTGGTAAGATCTTGAGAAAGGACTTGA

VvSTS	GAGCTAAGATCGCTTCTGGTGACTTGCCAAAGTAA<br>ATGGCTTCCGTTGAAGAATTCAGAAACGCTCAAAGAGCTAAA<br>GGTCCAGCTACTATTTTGGCTATTGGTACTGCTACTCCAGATCA<br>TTGTGTTTACCAATCTGATTACGCCGACTACTACTTCAGAGTTA<br>CTAAGTCTGAACACATGACCGAATTGAAGAAAAAGTTCAACA<br>GAATCTGCGACAAGTCCATGATCAAGAAGAGATATATCCACTT<br>GACCGAAGAAATGTTGGAAGAACATCCAAACATTGGTGCTTAT<br>ATGGCTCCATCCTTGAACATCAGACAAGAAATTATCACTGCCG<br>AAGTTCCAAGATTGGGTAGAGATGCTGCTTTGAAGGCTTTGAA<br>AGAATGGGGTCAACCTAAGTCTAAGATCACCCATTTGGTTTTC<br>TGTACTACCTCTGGTGTTGAAATGCCAGGTGCTGATTACAAATT<br>GGCTAACTTGTTGGGTTTGGAAACCTCCGTTAGAAGAGTTATG<br>TTGTACCATCAAGGTTGTTATGCTGGTGGTACTGTTTTGAGAAC<br>TGCTAAAGATTTGGCTGAAAACAATGCTGGTGCTAGAGTTTTG<br>GTTGTTTGCTCTGAAATTACCGTTGTTACTTTCAGAGGTCCATC<br>TGAAGATGCTTTGGATTCTTTGGTTGGTCAAGCTTTGTTTGGTG<br>ATGGTTCTTCTGCTGTTATAGTTGGTTCTGATCCAGATGTCTCTA<br>TCGAAAGACCTTTGTTCCAATTGGTTTCTGCTGCTCAAACTTTC<br>ATTCCAAATTCTGCTGGTGCAATTGCTGGTAACTTGAGAGAAG<br>TTGGTTTGACTTTTCATTTGTGGCCAAACGTTCCAACTTTGATC<br>TCCGAAAACATTGAAAAGTGTTTGACCCAAGCTTTCGATCCAT<br>TGGGTATTTCTGATTGGAATTCCTTGTTCTGGATTGCTCATCCA<br>GGTGGTCCAGCAATTTTGGATGCTGTTGAAGCTAAATTGAACT<br>TGGAAAAGAAGAAGTTGGAAGCCACCAGACATGTTTTGTCTG<br>AATACGGTAATATGTCCTCTGCTTGCGTTTTGTTCATTTTGGAC<br>GAAATGAGAAAAAAGTCCTTGAAGGGTGAAAAGGCTACTACT<br>GGTGAAGGTTTGGATTGGGGTGTTTTGTTCGGTTTTGGTCCAG<br>GTTTGACTATTGAAACTGTTGTCTTGCATTCTGTTCCAACCGTT<br>ACCAATTGA
